# Optimism, Social Identity, Mental Health: Findings Form Tibetan College Students in China

**DOI:** 10.3389/fpsyg.2021.747515

**Published:** 2021-10-14

**Authors:** Yongyong Chen, Jing Su, Yi Zhang, Wenfan Yan

**Affiliations:** ^1^School of Education, Qinghai Normal University, Xining, China; ^2^Institute of Plateau Science and Sustainable Development Research Team of Plateau Ethnic Psychology and Behaviour, Qinghai Normal University, Xining, China; ^3^Mental Health Center for College Students, Shandong Technology and Business University, Yantai, China; ^4^Institute of International and Comparative Education, University of Massachusetts Boston, Boston, MA, United States

**Keywords:** optimism, social identification, identification, mental health, health, minority students

## Abstract

Social identity runs through the whole life of an individual, and it provides a framework to help individuals form a value guide adapted to their survival and development in different social situations and multiple roles. This study aimed to explore the mediating effect of social identity on the relationship between optimism and mental health among 659 Tibetan college students in China. We used the Depression Anxiety and Stress Scales, the Satisfaction with Life Scale, and the Positive Affect Scale and developed a tool to assess optimism, which included three subscales measuring optimistic tendency, pessimistic tendency, and self-efficacy optimism. In addition, we have developed a social identity scale for Tibetan college students in China. Results indicated that the optimism of Tibetan college students in China had a significant positive impact on their mental health and that social identity can affect their optimism to further improve their mental health. These findings provide guidance for implementing psychological interventions aimed at enhancing undergraduates’ mental health.

## Introduction

A healthy psychological status is a *sine qua non* for the academic success and optimal development of minority college students. Previous research has demonstrated differences in the levels of physical and mental health among ethnic minority college students, compared to other students (Li and Liu, 2014; Wei et al., 2017). Due to pressure related to ethnic status, imposter syndrome, and stereotypes, among other factors, the mental health levels of African-American, Asian-American, and Latino American college students are lower than those of white college students (Cokley et al., 2013). The overall mental health level of minority college students in China is significantly lower than that of the general college student population nationwide (Gao et al., 2013), especially in the diagnosis of obsessive–compulsive symptoms, anxiety, and depression (Wu, 2019). Although the mental health of ethnic minority college students has been the focus of extensive work, both in China and elsewhere (Vacek et al., 2010; Xin and Liu, 2019), there are few studies on individual ethnic groups, and the sample sizes of most studies are very small (Guo et al., 2014).

Tibetans, one of the ethnic minorities in western China, comprise a total population of 6.28 million, accounting for a large portion of China’s minority population (China’s sixth census, 2011). As an elite group among their ethnic peers, Tibetan college students shoulder the important task of national construction and social development in China. However, their mental health prospects are not encouraging. Gao et al. (2015) selected 993 Tibetan college students from five universities in western China to investigate and analyze their mental health status and its influencing factors. The survey found that, among Tibetan college students in China, female students, second year university/college students, students from farming and pastoral areas, and students whose parents had low educational levels faced more serious psychological problems. This indicates that more attention should be paid to the mental health of Tibetan college students in China. In line with Wang and Zhang (2011) proposed two-factor structural model of mental health, mental health includes the acquisition of positive mental health and the elimination of negative mental health. As a result, for the purposes of this study, we have selected the corresponding positive and negative indicators.

Optimism is a long-term, cross-situational, and stable personality characteristic concerning one’s outlook on future positive or negative life events (Scheier and Carver, 1985). Optimism has been associated with improved physical health, enhanced happiness, and the promotion of professional success in individuals (Singh and Jha, 2013; Hao et al., 2016). Previous studies have found a significant positive correlation between optimism, social support, and positive health behavior among Asian-Americans aged 18 to 21, where optimism, as a mediator variable, affected the relationship between social support and positive health behavior (Ayres and Mahat, 2012). Due to differences in geographical location, religious culture, and customs, the personality traits of Tibetan college students in China show both communality and individuality. Traditionally, Tibetan people enjoy group activities and have many cultural ceremonies – customs that are conducive to the formation of the generally outgoing and sociable personalities of Tibetan college students in China (Wang, 2012). At present, researchers have different views on the structure of optimism. They first thought optimism was a one-factor structure (Scheier and Carver, 1985), some researchers put forward that optimism and pessimism in optimism are independent of each other later (Marshall et al., 1992), that is, optimism is divided into two dimensions: optimism and pessimism. In addition, some researchers believe that optimism can be manifested in different areas of individual life, including personal optimism, social optimism, and self-efficacy optimism (Schweizer and Koch, 2001). In order to discuss the impact of optimism on mental health in more detail, this study adopts a multi-dimensional optimism structure, that is, optimism is divided into optimistic tendency, pessimistic tendency, and self-efficacy optimism. Optimistic tendency means that individuals tend to evaluate the development trend and consequences of things positively, and pessimistic tendency means that individuals evaluate the development trend and consequences of things negatively. Self-efficacy optimism is the expectation of one’s own behavior results, which has nothing to do with one’s previous behavior experience. It is an integral part of optimism.

This study primarily adopted the multi-dimensional structure of optimism to explore its impact on mental health. Optimism was divided into optimistic tendency, pessimistic tendency, and self-efficacy optimism (Chen and Huo, 2016). The optimistic tendency in this study referred to the individual’s tendency to positively evaluate the developmental trends and consequences of things, while the pessimistic tendency referred to the individual’s negative evaluation of the developmental trends and consequences of things. Self-efficacy optimism, specifically, is the expectation of the results of one’s own behavior, which has nothing to do with one’s previous behavioral experience.

Social identity is an important subject in the field of social psychology. Tajfel (1978) proposed that social identity means that an individual realizes that they belong to a specific social group, and at the same time realize the emotional and value significance received as a group member. Western studies of social identity have proliferated, yielding useful results. Studies indicate that social identity can be conducive to the functioning and harmonious development of society (Haslam and Platow, 2001). Social identity and life satisfaction were both found to be significantly positively correlated with each other, and negatively correlated with stress (Haslam et al., 2005). In addition, some studies have demonstrated significant differences in the social identity structure of minority college students, compared to other students, with a significant correlation between the number of social identity characteristics and their self-esteem. Individuals with high multiple social and personal identities have the highest self-esteem; for example, three social identity characteristics are found in African-Americans, four social identity characteristics in Asian-Americans, and two social identity characteristics in Latin-Americans (Gonzales-Backen et al., 2015). However, this field of inquiry is relatively young in China, with studies focusing mainly on floating populations and special groups (Yan, 2016). Only a few studies have focused on the social identity of minority college students (Jiang and Li, 2011), and almost no studies have focused on specific minority groups.

Therefore, when individuals highly recognize a certain group and society, it is not only conducive to their own development, but also to the normal and healthy operation of society at large. Through combing and summarizing the literature, it is found that the structure of social identity in China mainly consists of four dimensions, that is, the dimension of cognition, emotional, motivation, and behavioral. According to research needs, four dimensions of social identity are adopted as: belonging, emotional, evaluation, and behavioral. Belonging identity is one’s perception of one’s group, including the self-understanding of the members of the group, such as enjoying being with Tibetan students and being interested in their own history. Emotional identity refers to the negative or positive emotions of individuals after they join the group, for example, happiness about being Tibetan; liking their hometown and grassland; preferring Tibetan to Han lifestyle; and believing that Tibetan compatriots have many excellent qualities, such as kindness, bravery, singing, and dancing skills. Behavioral identity refers to the emotional dependence and connection of an individual to his/her group, including the behavioral tendency or activities carried out in order to maintain the interests, culture, and identity of the group; for example, striving to inherit the Tibetan language and culture; understanding the national taboos and not breaking them; studying to contribute to the improvement of his/her hometown; and returning to their hometown to work after graduation. Evaluation identity is the evaluation of significance of sharing and understanding social values, including not only the evaluation of other groups, but also the consensus reached by individuals and members of their groups. Therefore, social identity is conducive to the development of the individuals and the normal operational health of society at large. This paper hypothesizes that among Tibetan college students in China, there is a correlation between optimism, social identity, and mental health (hypothesis 1).

Optimism, according to previous studies, has a significant positive correlation with positive indicators of mental health and is negatively correlated with negative indicators of mental health (Zhou et al., 2015). During adaptation to a new environment, optimism can exert a positive impact, providing an effective barrier against frustrations and stress related to adversity in life. As an important indicator of mental health levels, college students’ optimism can have a critical role in their healthy development. Yi (2012a,b) argued that the tendency of college students to be optimistic had a moderate negative correlation with depression symptoms, which means that an increase in optimistic emotions can effectively relieve negative emotions at a mental health level. Therefore, this paper hypothesizes that the optimism of Tibetan college students in China has a positive impact on their mental health level (hypothesis 2).

Furthermore, studies have shown that the mental health of Chinese minority college students is highly related to their social identity (Wang et al., 2006). National identity and optimism are positively related, and national identity is an important part of social identity (Kong, 2014). This indirectly demonstrates that social identity and optimism are positively related. Thus, we hypothesize that for Tibetan college students in China, there is a mediating effect of optimism on the relationship between mental health and social identity (hypothesis 3).

Although some researchers have explored the mechanisms by which optimism affects mental health (Conversano et al., 2010; Qi et al., 2012; Zhou et al., 2015; Rincón Uribe et al., 2020), few studies have explored the mediating role of social identity between optimism and mental health based on self-concept. This study explores the affective mechanisms underlying these variables to further analyze factors affecting the mental health of Tibetan college students in China, and provide guidance for enhancing the mental health of ethnic minority college students.

## Materials and Methods

### Participants

College students from four universities (Qinghai Normal University, Northwest University for Nationalities, Qinghai University for Nationalities, and Tibet University) were recruited through a stratified cluster random sampling method. All participants are voluntary and have obtained informed and consent before completing the test. Their names were not collected and they were assured of the confidentiality of their responses. A total of 680 questionnaires were sent out and 659 valid questionnaires were retrieved (effective recovery rate=96.91%). In the sample, 56.47% were female, 43.09% were male, and 0.44% did not report their sex. Participants were between 17 and 25years old (*M*=21.27, *SD*=1.15).

### Measures

#### Optimism Scale of Tibetan College Students in China

An exploratory factor analysis showed that the 23-item Optimism Scale comprises three dimensions: optimistic tendency (seven items), pessimistic tendency (six items), and self-efficacy optimism (10 items). AMOS 22.0 was used to investigate the validity of the model through a confirmatory factor analysis. The results showed as: *χ*^2^/*df*=4.06, goodness of fit index (GFI)=0.87, adjusted goodness of fit index (AGFI)=0.91, root mean square error of approximation (RMSEA)=0.071, comparative fit index (CFI)=0.93, and root mean square residual (RMR)=0.082, which indicated good construct validity and that the three-factor model was an adequate fit for the data. A five-point Likert self-evaluation scale was used ranging from 1 (*high non-conformity*) to 5 (*high conformity*). The overall internal consistency coefficient of the questionnaire was 0.835, and the split-half reliability was 0.787. Four weeks after the initial questionnaire completion, 62 students from Qinghai Normal University were retested, and the retest reliability was calculated; the overall internal consistency coefficient of the questionnaire reached 0.643, meaning that the questionnaire had strong reliability as a research measurement tool. The Cronbach’s *α* coefficient of the scale was 0.84.

#### Social Identity Scale of Tibetan College Students

An exploratory factor analysis showed that the 25-item Social Identity Scale comprises four dimensions: belonging identity (six items), emotional identity (nine items), behavioral identity (five items), and evaluation identity (five items). AMOS 22.0 was used to perform confirmatory factor analysis to further validate the model. The results showed that *χ*^2^/*df*=4.14, GFI=0.89, AGFI=0.90, RMSEA=0.064, CFI=0.91, RMR=0.085, which indicated that the four-factor model had reliable data and construct validity. A five-point Likert self-evaluation scale was used ranging from 1 (*high non-conformity*) to 5 (*high conformity*). The overall internal consistency coefficient of the questionnaire was 0.862, with a split-half reliability of 0.842. Four weeks later, 62 students from Qinghai Normal University were retested to recalculate reliability; the overall internal consistency coefficient of the questionnaire reached 0.663, which shows that the questionnaire has strong reliability and could be used as a research measurement tool. The Cronbach’s *α* of the scale was 0.84.

#### Brief Version of the Depression Anxiety Stress Scales (DASS-21)

The Chinese version of the DASS-21 revised by Gong et al. (2010) was used in the study. The DASS-21 consists of three subscales to measure depression, anxiety, and stress, each of which contains seven items. The scale uses a 4-point Likert scoring format (from “not at all” to “very consistent”). The internal consistency coefficient for the total scale was 0.89; the internal consistency coefficient for the depression subscale was 0.77, and the anxiety subscale was 0.79. Two subscales in the DASS-21 for depression and anxiety were used as negative mental health indicators; higher scores indicated stronger emotions. Consequentially, the *α* coefficients for depression and anxiety were 0.84 and 0.81, respectively. The Cronbach’s *α* for the scale was 0.83.

#### Satisfaction With Life Scale

The five-item Satisfaction with Life Scale (SWLS), developed by Diener et al. (1985), evaluates university students’ life satisfaction. Items are scored on a seven-point Likert scale ranging from 1 (*strongly disagree*) to 7 (*strongly agree*); the higher the score, the higher the students’ satisfaction with life. Pavot and Diener (1993) found that for the SWLS, the internal consistency coefficient was 0.87 and the two-month follow-up coefficient was 0.82, which indicates a strong criterion validity. Moreover, the correlation between the SWLS and other subjective health scores indicated that this scale had good content validity. In this study, the Cronbach’s *α* of the scale was 0.82.

#### Positive Affect Scale

The Positive Affect Scale (PAS) was selected from the positive emotion subscale of the positive and negative emotions scale compiled by Watson et al. (1988). It consists of 10 items scored on a five-point Likert self-assessment scale ranging from 1 (*almost no emotions*) to 5 (*extremely many emotions*). A higher score correlates with higher positive emotions. Huang et al., (2003) showed that the inter-rater consistency for the positive emotion scale was 0.85, which means that the PAS has strong reliability. In this study, the Cronbach’s *α* coefficient of the scale was 0.88.

#### Common Method Bias Test

As this study relies on self-reports for data collection, a common method bias effect might exist (Zhou and Long, 2004). Thus, we employed multiple methods to control for bias: (1) all questionnaires were anonymous; (2) the scales and questionnaires had relatively high reliability and validity to mitigate systematic errors in measurement; (3) some questionnaire items were scored through reverse scoring; and (4) participants were from four different schools, which increased the differences caused by different regions. In addition, after data collection, Harman’s single-factor test was used to diagnose the common method deviation. The results showed that the eigenvalues of five factors were greater than those without rotation, and the variance of the first-factor interpretation was 25.62%, less than the critical standard of 40%. This shows that the common method bias effect was not obvious.

### Procedure

The questionnaire administrators were trained ethnopsychology and developmental psychology graduate students with prior experience in conducting surveys. The test was conducted in a quiet classroom environment and administered in small class groups. Before the formal start time, the tester read the test instructions, requirements, and other related questions to all the participants loudly and clearly. The participants completed the test in approximately 30min and the questionnaires were immediately collected. The questionnaires were screened; blank or regular questionnaires were eliminated, and AMOS 20.0 was used to analyze the remaining data.

## Results

### Correlation Between Optimism, Social Identity, and Mental Health of Tibetan College Students in China

Pearson’s correlation analysis was used with the three variables of optimism, social identity, and mental health of Tibetan college students in China. The results are shown in Table 1. For Tibetan college students in China, the correlation coefficients between optimism (optimistic tendency, pessimistic tendency, and self-efficacy optimism), social identity (belonging identity, emotional identity, behavioral identity, and evaluation identity), and mental health (positive and negative) were all high and had strong relative stability (Table 1). These findings provide preliminary support for further hypothesis testing.

**Table 1:** Correlation analysis between variables.

S.no	*M*	*SD*	Optimism			Social Identity				Mental Health			
			1	2	3	4	5	6	7	8	9	10	11
			Optimistic tendency	Pessimistic tendency	Self-efficacy optimism	Belonging identity	Emotional identity	Evaluation identity	Behavioral identity	Anxiety	Depression	Life satisfaction	Positive emotion
1	3.611	0.556	–										
2	2.984	0.551	−0.099^*^	–									
3	3.310	0.524	0.530^***^	−0.321^**^	–								
4	4.976	0.783	0.257^***^	−0.064^*^	0.130^**^	–							
5	5.293	0.884	0.367^***^	−0.049^*^	0.166^**^	0.542^**^	–						
6	5.946	1.364	0.280^***^	−0.092^**^	0.143^**^	0.546^**^	0.482^**^	–					
7	5.946	1.364	0.423^***^	−0.037	0.252^***^	0.475^**^	0.587^**^	0.553^**^	–				
8	0.067	0.585	−0.234^**^	0.312^***^	−0.072^*^	−0.255^**^	−0.340^**^	−0.291^**^	−0.342^**^	–			
9	0.745	0.565	−0.140^**^	0.320^***^	−0.096^*^	−0.216^**^	−0.288^**^	−0.247^**^	−0.295^**^	0.535^**^	–		
10	4.260	1.140	0.323^***^	−0.061^*^	0.331^***^	0.159^**^	0.220^**^	0.210^**^	0.238^**^	−0.144^**^	−0.150^**^	–	
11	3.110	0.608	0.346^***^	−0.093^*^	0.443^***^	0.087^*^	0.143^**^	0.138^**^	0.198^**^	−0.029	0.010	0.426^**^	–

* *p<0.05*;

** *p<0.01*;

*** *p<0.001*.

### Multiple Regression Analysis of Optimism on Mental Health Level

To test the influence of optimism on the mental health of Tibetan college students in China, the study conducted regression analysis by using every dimension of optimism as an independent variable and the (positive and negative) mental health level of Tibetan college students in China as a dependent variable. Results showed that all dimensions of optimism had significant positive predictive effect on positive mental health (life satisfaction and positive emotions), and all dimensions of optimism had significant negative predictive effect on negative mental health (depression and anxiety). The total variance explained in mental health was 20.0%, *F*(4, 654)=63.506, *p*<0.001.

Pessimistic tendency had a significant positive predictive effect on positive mental health (life satisfaction and positive emotions), and all dimensions of optimism had a significant negative predictive effect on negative mental health (depression and anxiety). The total variance explained in mental health was 18.5%, *F*(4, 654)=49.830, *p*<0.001.

Self-efficacy optimism had a significant positive predictive effect on positive mental health (life satisfaction and positive emotions), and each dimension of optimism had a significant negative predictive effect on negative mental health (depression and anxiety). It explained 23.3% of the total variance in mental health, *F*(4, 654)=77.242, *p*<0.001 (Table 2).

**Table 2:** Multiple regression analysis of optimism to mental health.

Dependent variable	Independent variable	*R* ^2^	*F*	*β*	*t*	SS	MS
Life satisfaction	Optimistic tendency	0.200	63.506	0.091	5.742^***^	68.113	17.028
Positive emotions				0.241	8.148^***^		
Depression				−0.341	−6.793^***^		
Anxiety				−0.170	−3.266^***^		
Life satisfaction	Pessimistic tendency	0.185	49.830	−0.103	−2.173^**^	43.516	12.379
Positive emotions				−0.126	−2.870^**^		
Depression				0.147	2.871^**^		
Anxiety				0.182	3.406^***^		
Life satisfaction	Self-efficacy optimism	0.233	77.242	0.078	5.492^***^	65.428	16.357
Positive emotions				0.326	12.419^***^		
Depression				−0.107	−2.408^**^		
Anxiety				−0.137	−2.963^**^		

^*^p<0.05;

** *p<0.01*;

*** *p<0.001*.

### Optimism Affecting Mental Health Level: Mediating Effect Model of Social Identity

According to our research results, we posit a mediation model to explore the internal mechanism of optimism that affects mental health, by taking social identity as the mediator, optimism as the independent variable, and mental health level as the dependent variable. This study constructed three models with the fit index, which had acceptable ranges for further analysis of the results (Table 3). First, in model A, optimism affects positive mental health through social identity (Table 3A). Second, in model B, optimism affects and focuses on the level of negative mental health through social identity (Table 3B). Third, in model C, optimism affects and focuses on the level of mental health through social identity (Table 3C). This study adopted the Bootstrap method proposed by Hayes, (2013) to test the mediation effect by selecting a sample size of 5,000.

**Table 3:** Fit index of each model.

Model	*χ*^2^/*df*	RMSEA	CFI	IFI	TLI	NFI
A	2.584	0.039	0.946	0.946	0.933	0.915
B	2.419	0.037	0.952	0.952	0.942	0.921
C	2.492	0.039	0.937	0.937	0.933	0.900

*RMSEA, root mean square error of approximation; CFI, comparative fit index; IFI, incremental fit index; TLI, Tucker–Lewis index; and NFI, normed fit index*.

Model A had good fit with the data. Specifically, optimism played a significant and positive predictive role in social identity and positive mental health. Social identity can significantly predict positive mental health. Optimism had a direct and intermediary effect on positive mental health. The direct effect of optimism on positive mental health was 0.552, which accounted for 90.05% of the total effect of 0.613. The mediating effect was 0.061, which accounted for 9.95% of the total effects of 0.613. The results of bootstrap analysis showed that the 95% confidence interval of “optimistic → social identity → positive mental health” did not contain 0, which verified social identity had a partial mediating role in the relationship between optimism and positive mental health.

Model B had a good fit with the data as well. Specifically, optimism played a significant and positive predictive role in social identity, whereas social identity significantly and negatively predicted negative mental health. The path coefficient from optimism to negative mental health was not significant. According to the Sobel test, social identity had a significant role in mediating optimism and negative mental health, especially *Z*=2.309. In addition, the results of bootstrap analysis showed that the 95% confidence interval of “optimistic → social identity → negative mental health” did not contain 0, which verified social identity has a full mediating role in the relationship between optimism and negative mental health.

**Figure 1:**
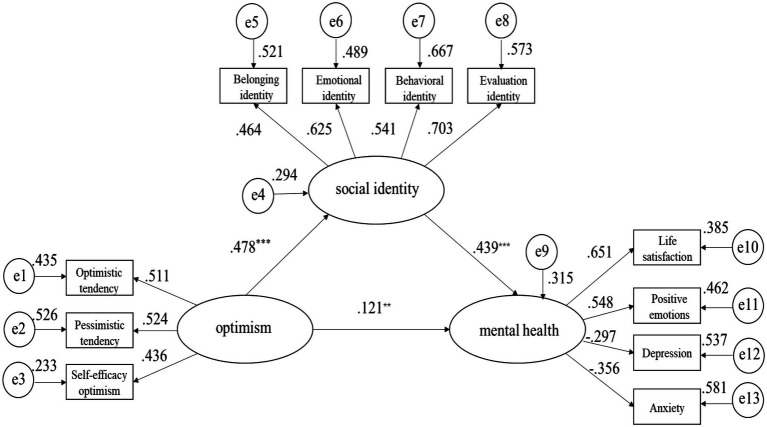
Relationship model and standardized path coefficient of optimism, social identity, and mental health. ^**^p<0.01; ^***^p<0.001.

Model C also had good fit with the data. Specifically, optimism played a significant and positive predictive role in social identity and mental health, whereas social identity significantly and positively predicted mental health. The direct effect of optimism on mental health was 0.121, which accounted for 36.56% of the total effect of 0.331. The mediating effect was 0.210, which accounted for 63.44% of the total effect. The results of bootstrap analysis showed that the mediating effect did not include 0 [lower level confidence interval (LLCI)=0.0052, upper level confidence interval (ULCI)=0.0862] with the 95% confidence interval indicating the significant mediating effect of social identity. After controlling for the social identity of the mediating variable, the impact of optimism on mental health was still significant, but the interval (LLCI=0.1133, ULCI=0.3342) did not include 0. Thus, the relationship between optimism, social identity, and mental health level of Tibetan college students in China effectively supported the mediating effect model. The mediation model diagram and standardized path coefficient of the study are displayed in Figure 1.

## Discussion

The results showed that the correlations between optimism, social identity, and mental health level were relatively high among Tibetan college students in China, verifying Hypothesis 1. This shows that optimism, social identity, and mental health level are closely related, making it possible to further study the deep influential mechanisms underlying these relationships.

Although existing research has shown the significant role of optimism in predicting mental health, this study added the dimensions of optimism, that is, self-efficacy optimism, and constructed a regression model between optimistic tendency, pessimistic tendency, self-efficacy optimism, and mental health to explore the impact of the three dimensions of optimism on mental health in more detail. The results showed that optimism has a positive impact on the mental health of Tibetan college students in China, which verifies hypothesis 2.

This study considers social identity as the intermediary in constructing a structural equation model on the effect of optimism on the positive mental health level of Tibetan college students in China. In line with our initial expectations, optimism can directly affect the mental health level of Tibetan college students in China while indirectly affecting their mental health level through social identity. In the face of the impact of different ethnic cultures and the collision of life styles, optimistic individuals have a positive attitude toward life and are more willing to use effective coping strategies than pessimistic individuals, constantly adjust their own state, and strive to integrate into the new environment. They are better at maintaining social networks and close relationships, so optimistic individuals can get more social support (Wang, 2008). Furthermore, it increases the social identity of the group one belongs to and stimulates the positive emotions of the members of a group (Junker et al., 2019). Social identity is a way to enhance psychological wellbeing. Through the realization and maintenance of positive social identity, individuals can enhance their sense of belonging, improve their life satisfaction, and find the significance of being alive. The results of the current research showed that the more optimistic Tibetan college students in China are, the higher their social identity will be, which is beneficial for them to cope with problems in academics and life more confidently and to maintain a more positive self-evaluation.

Taking social identity as the intermediary, this study constructed a structural equation model for the effect of Tibetan college students’ optimism on their mental health level in China. Studies have found that social identity has a full mediating role in the relationship between optimistic tendency and anxiety and between self-efficacy optimism and depression. However, it has a partial mediating role in other relationships. Our results are consistent with previous research on social identity. Nesdale et al. (1997) proposed that people might be unable to adapt to new cultural environments because of cultural conflicts, which generates anxiety. Research on ethnic minority college students in China also shows that they are susceptible to psychological adjustment difficulties and psychological symptoms, such as depression, anxiety, and loneliness (An et al., 2019). Social identity is an important intermediary variable, which is related to the individual’s psychosocial adaptation level. Individuals with low social identity lack self-confidence in their study and life, and they also adopt passive and evasive ways in interpersonal communication, which seriously affects their psychological adaptation level in the university environment, leading to depression, anxiety, and other psychological problems. High levels of social identity can alleviate the negative effects of cultural shock on teenagers, such as problem behavior and anxiety, and provide a kind of protection and buffer. Hypothesis 3 is thus verified. Therefore, the construction of positive social identity is of great significance to the healthy development of Chinese minority youth. Thus, we should emphasize the cultivation of optimism in minority college students in China and the development of their social identity to help improve their psychological adaptability and mental health.

In multi-ethnic countries, identity confusion and cultural adaptation are often more obvious among ethnic minorities or vulnerable groups. As a large ethnic group in China, Tibetans have relatively fixed settlements (Tibet, Qinghai, Sichuan, Gansu, etc.). Before entering colleges and universities, they are in contact with the members of their own ethnic group and are immersed in their own culture. To pursue higher education, Tibetan college students in China leave their hometowns to enter more diversified social environments. In this way, they face the task of adaptation to different ethnic cultures and lifestyles, in addition to the growth and development tasks faced by other college students. On the one hand, they need to behave consistently with their own customs and habits while, on the other hand, experiencing and integrating the impact of other cultures and contexts. This may aggravate internal contradictions, leading to adverse reactions, such as anxiety and depression, and otherwise affecting their mental health (Li, 2009). This study of social identity sheds light on “identity confusion” among Tibetan college students in China, which can be useful in improving their sense of happiness. A high degree of social identity yields higher social cohesion and can be a critical soft power tool for the promotion of national development. In other words, to explore, the impact of social identity on the mental health level of Chinese ethnic minority college students is not only conducive to promoting their psychological harmony, but also affects the integrative and society-building attitudes and behaviors of this group, helping to avoid social development risks and effectively increasing the speed of social development (Jiang and Li, 2011). A positive social identity, thus, is conducive to the development of national unity and social stability in geographic areas with a high number of ethnic minorities.

Our findings are also an important inspiration for other relevant lines of research. Like Tibetan college students in China, minority college students in other multi-ethnic countries are also faced with the dilemma of identity and the test of cultural adaptation. For example, in the United States, African-Americans (Atlanta) and Asian-Americans (San Francisco) all have relatively stable residences. To enhance their development, they moved from their communities and cities of origin to a society dominated by the mainstream culture. When facing the impact of different cultures, social identity guides ethnic minorities to determine what is valuable and how to navigate diverse roles and social situations. The findings of this study can not only be used to interpret the construction and development of individual and social identity of minority college students studying in China, but can also be extended to the construction of social identity of minority college students in the global perspective. This is a study on the issue of national education in China based on western theories and is also an attempt to enrich and develop western social research theories by educational practice in China, which expands the space for studying social problems and social phenomena at home and abroad. Therefore, it has reference and application value for many problems in the field of education all over the world. Tracing back to the origin of social identity theory demands interpreting the behavioral ethnocentrism among different populations. Optimism, as an important concept in positive psychology, was combined with social identity in the present research to show that social identity has a mediating effect on the relationship between optimism and mental health level of Tibetan college students in China. It not only deepens and expands social identity theories, but also offers a new thread and method to relevant global research on social identity. It provides reference and experience for other multi-ethnic countries, and valuable guidance for promoting the wellbeing of all mankind.

This study has some limitations. First, it adopted a psychometric method to collect data. Thus, the collected data may have the effect of standard method variance and social desirability bias. However, to mitigate the adverse effects of the psychometric method, our optimism and social identity questionnaires were created with high reliability and validity. Additionally, we used exploratory and confirmatory factor analyses to ensure a highly reliable internal validity. However, for improvements to the research design and to further validate the results, follow-up studies should utilize a tracking data analysis method. Second, this paper primarily focused on Tibetan college students in China, failing to compare it with the dominant ethnicity, Han college students, as the control group. Hence, future research should be conducted to further reveal the differences between the two groups. Third, further studies are still required on the mechanism of effect of social identity on the mental health level of Tibetan college students in China. This study only analyzed the mediating role of social identity in optimism and mental health. Future research should consider additional core factors. In the related analysis of social identity, research on the identity or separation attitude of mainstream cultural groups should be explored.

## Conclusion

Our study found that the optimism of Tibetan college students in China has a significant positive impact on their mental health level. The effect of optimism and social identity on the mental health of Tibetan college students in China conformed to the mediating effect model. Social identity was found to have a partial mediating effect on the relationship between optimism and mental health level. Therefore, social identity has a partial mediating role in the relationship between optimistic/pessimistic/self-efficacy optimism and positive mental health (life satisfaction/positive emotions). Social identity has a partial mediating role in the relationship between optimistic tendency and depression, pessimistic tendency and anxiety/depression, and self-efficacy optimism and anxiety, but a full mediating role in the relationship between optimistic and anxiety, as well as between self-efficacy optimism and depression.

## Data Availability Statement

The data analyzed in this study are subject to the following licenses/restrictions because the research subject is a Chinese Tibetan college student. Requests to access these datasets should be directed to YC, chenyongyong@qhnu.edu.cn.

## Ethics Statement

All procedures performed in studies involving human participants were in accordance with the ethical standards of the institutional research committee and with the 1964 Helsinki Declaration and its later amendments or comparable ethical standards. All participants provided their written informed consent to participate in this study.

## Author Contributions

All authors contributed to the study conception and design. Material preparation, data collection, and analysis were performed by YC, JS, YZ, and WY. The first draft of the manuscript was written by YC and all authors commented on previous versions of the manuscript. All authors read and approved the final manuscript.

## Funding

This research was supported by the National Office for Philosophy and Social Sciences, Pedagogy area, Youth theme, “The Dynamic evaluation and promote research on Qinghai minority college students’ positive psychological quality and mental health level” (CMA170246).

## Conflict of Interest

The authors declare that the research was conducted in the absence of any commercial or financial relationships that could be construed as a potential conflict of interest.

## Publisher’s Note

All claims expressed in this article are solely those of the authors and do not necessarily represent those of their affiliated organizations, or those of the publisher, the editors and the reviewers. Any product that may be evaluated in this article, or claim that may be made by its manufacturer, is not guaranteed or endorsed by the publisher.
